# Pleotropic effects of statins: the dilemma of wider utilization of statin

**DOI:** 10.1186/s43044-023-00327-8

**Published:** 2023-01-05

**Authors:** Ambika Choudhary, Ujjawal Rawat, Piyush Kumar, Piyush Mittal

**Affiliations:** Teerthanker Mahaveer College of Pharmacy, Teerthanker Mahaveer University, Moradabad, India

**Keywords:** Statins, Cardiovascular diseases, Atherosclerosis, Pleotropic effects, Cancer

## Abstract

**Background:**

Apart from reducing the circulating LDL-c and the number of cardiovascular cases as well as fatalities, statins have auxiliary non–lipid-related or cholesterol independent effects, the pleiotropic effects. The aim of the present review is to understand the pleotropic effects of statins.

**Main body:**

Cardiovascular disease (CVD) is presently the major cause of patient misery as well as mortality among non-communicable diseases (NCDs) in the world. Despite the fact that statins are the most extensively affirmed, prescribed and evidence-based lipid-lowering medicine worldwide that curtail low density lipoprotein cholesterol (LDL-c) levels and the number of cardiovascular cases as well as deaths, statins also elicit auxiliary non–lipid-related or cholesterol independent effects, the pleiotropic effects. Improved endothelial function, significantly lowered oxidative stress, atherosclerotic plaque stabilization, immunomodulatory, cessation of vascular smooth muscle proliferation, effects on bone metabolism, anti-inflammatory, antithrombotic effects, and reduced risk of dementia are among these pleotropic effects. Statins have also been explored for its uses in life threatening diseases like cancer and inflammatory bowel disease. They have been demonstrated to revamp vascular tone. Many research and review articles have been thoroughly studied for this systematic review.

**Conclusions:**

Statins have not only shown to be benefitial in lowering the levels of LDL-C but have also been established to be advantageous in the treatment of cancer, neurological conditions like dementia, multiple sclerosis, inflammatory bowel disease. Future high-quality trials are needed to include statins in the treatment of these conditions as per guidelines.

## Background

The epidemiological switch in the twentieth century was abetted by a decrease in communicable disease mortality and disability and an expansion in noncommunicable disease deaths and disability (NCDs). Cardiovascular disease (CVD) is presently the major cause of patient suffering as well as mortality among NCDs in the world [1]. According to WHO estimates, cardiovascular diseases are responsible for approximately 18.6 million deaths, which stands for 32% deaths around the globe, out of which one third of people i.e., 85% are under the age of 70 years surprisingly [2]. In addition, the number of people suffering from cardiovascular disorders has almost doubled, rising from 271 million in 1990 to 523 million in 2019 [3]. In Asian population, cardiovascular diseases are accountable for double the number of casualities caused by malaria, tuberculosis and HIV together [4]. Out of all the deaths caused by cardiovascular diseases, atherosclerotic cardiovascular diseases make up the majority and on analyzing the number of deaths because of various cardiovascular diseases, it was revealed that heart attack and stroke account for 4 out of every 5 deaths i.e., nearly a fifth of the total deaths globally [5].

As Atherosclerotic cardiovascular disease (ASCVD) peril and cholesterol level in blood have a well-entrenched articulation, if the load of atherogenic lipoprotein in blood is considerably diminished, the progression of atherosclerosis can be lowered and eventually reversed [6–8] and there has been persuasive evidence that lowering LDL-c can mitigate development of atherosclerotic cardiovascular disease [9]. Hence, to reduce the population suffering from cardiovascular diseases, and provide both primary as well as secondary prevention, statins are the most extensively affirmed, prescribed and evidence-based lipid-lowering medicine worldwide that curtail LDL-c and eventually reduce cardiovascular morbidity and mortality [10–12].

Aside from lowering the levels of cholesterol in circulation, statins have some supplementary non-lipid effects. They have been demonstrated to ameliorate vascular tone. HMG-CoA reductase inhibition boosted endothelial nitric oxide (NO) synthase activity in cell experimentations, leading to increased NO bioavailability, a key regulator of vascular smooth muscle cell (SMC) proliferation, vascular tone and platelet aggregation which is a key driver in plaque progression [13, 14]. Additionally, statins inhibit it by downregulating the proliferation as well as migration of SMC’S present in vasculature. Another important role of statins has also been linked to a reduction in platelet aggregation and exerting antithrombotic actions, both of which contribute to a general decrease in cardiovascular mortality. This finding is supported by the study conducted by Sikora et al. [15].

Not only it reduces platelet aggregation but also diminishes platelet adhesion. All these properties are attributed to alterations in the cholesterol to phospholipid ratios in platelets which result in increased pro-adhesion receptor density and higher expression of thromboxane A2 synthesis. Reduction in oxidative stress and anti-inflammatory characteristics are two other prominent pleiotropic effects of HMG-CoA reductase inhibitors [16].

Statins lower the levels of C reactive protein levels besides blocking inflammatory mediators like interleukin (IL) 1b and tumor necrosis factor (TNF)-alpha. These anti-inflammatory properties are crucial in avoiding the formation of atherosclerotic plaques.

## Main text

### Impact of statins on atherosclerotic plaque

#### Plaque regression

Plaque regression entails eliminating the lipid as well as necrotic core of the plaque, recovering endothelial function, and stopping the proliferation of intravascular smooth muscle cells [17].

**Fig. 1:**
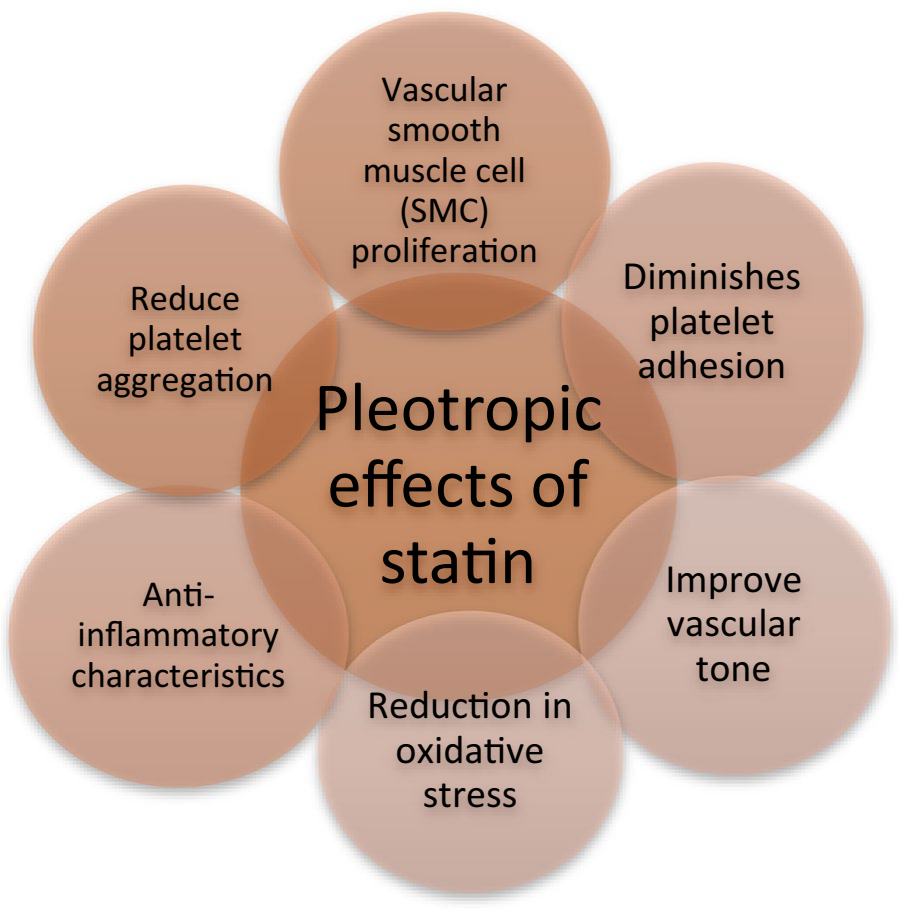
Pleotropic effects of statin

**Fig. 2:**
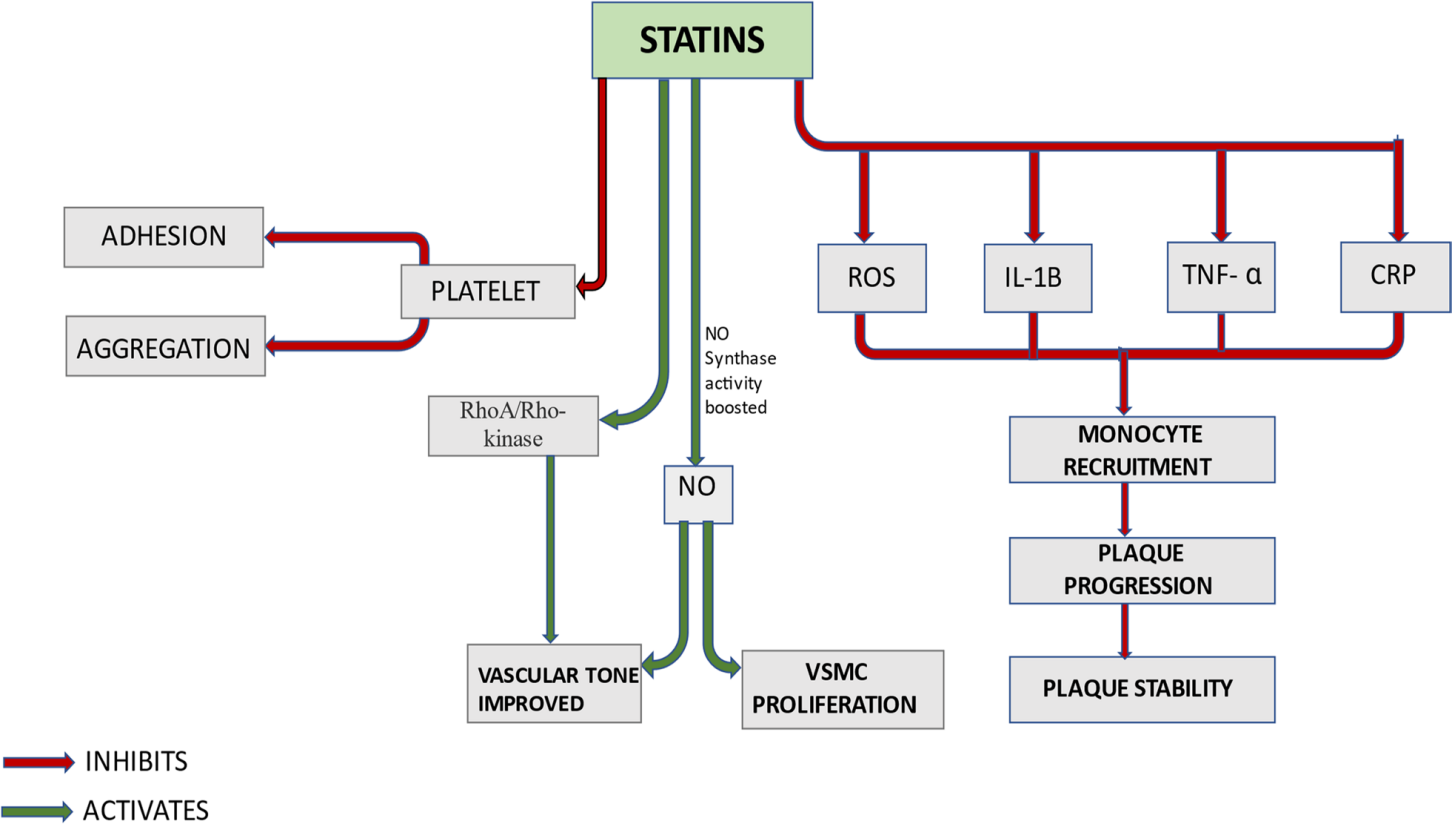
Role of statins in atherosclerosis (*NO* nitric oxide, *ROS* reactive oxygen species, *IL* 1B-interleukin 1B, *TNF-α* tumor necrosis factor α, *CRP* c reactive protein, *VSMC* vascular smooth muscle cell)

In the late 1990s, Brown et al. found that men with coronary artery disease (CAD) and a significant threat of following cardiovascular incidents who were managed with lipid-lowering therapy of any kind, spanning from statins to bile acid binders and niacin, had a 73 percent (95 percent CI 23–90 percent) lower incidence of clinical events and decelerated the advancement of coronary lesions [18]. The ASTEROID (Effect of Rosuvastatin Therapy on Coronary Artery Stenoses Assessed by Quantitative Coronary Angiography) trial also showed evidences of plaque regression (Table 1; Figs. 1, 2).

**Table 1:** Effect of statin on different organs of the body

Organ	Author	Summary
Liver	Foster et al	Combination regimen of vitamin E 1000 IU, vitamin C 1 g and atorvastatin 20 mg reduced relative risk of developing hepatic steatosis by 71% with a baseline NAFLD as compared to placebo
	Chomphupan et al	A low dose (10 mg) of Simvastatin is helpful in preventing stroke in type-2 diabetes patients
Kidney	Nayan et al	Statin utilization among kidney cancer patients is linked to higher survival rates
Nervous system	Salagre et al	If statins and anti-depressive therapy are used as combination therapy, symptoms of depression can be reduced significantly
	Jenson et al	Statins have beneficial effects in chronic inflammatory disorders like multiple sclerosis and optic neuritis
Bone	Nabarawi et al	Atorvastatin increase bone formation. Elevation of biomarkers of bone formation was seen in the serum. Apart from this, the bone reabsorption is also diminished
	Lin et al	Statin use was linked with a lower incidence of vertebral and hip fracture in stroke patients, and osteoporosis
	Gupta et al	Simvastatin has the ability to reduce periodontal infection,diminsh inflammation in the temporomandibular joint, improve alveolar and periodontal bone regeneration, cartilage repair and soft tissue grafting
	Thabit et al	Simvastatin or Atorvastatin treatment promoted bone mineral density
Lungs	Papaporfyriou et al	Using statins fluvastatin and atorvastatin, can lower levels of c-reactive protein, pulmonary hypertension, and mortality risk in people with COPD
	Mullerova et al	Statins may have antiviral and anti-inflammatory properties (down-regulation of cytokines)

Nissen et al. conducted a randomized trial in which he administered 40 mg pravastatin (moderate intensity) to one group while 80 mg atorvastatin (high intensity) to another group and derived that the group on atorvastatin did not have coronary atherosclerosis progression whereas an increase in atheroma volume was seen in the other group. Therefore, the intensity of statin therapy affects the progression of disease [19]. Multiple studies showed how significantly the statin therapy decelerated the rate of plaque progression [20, 21].

#### Plaque stabilization

Coronary artery calcification has gained popularity as a more reliable as well as accurate predictor of cardiovascular risk than conventional Framingham risk scores [22]. Although self-contradictory, but statins stabilize the plaque and amend the composition of atheroma by decreasing non calcified plaque and escalating the fraction of dense calcified plaque. In vascular calcification, different macrophage phenotypes play a significant role. The two phenotypes of macrophages work in the opposite way. As the M1 phenotype is responsible for advancement of atherosclerosis, plaque stabilisation relies heavily on the M2 phenotype [23].

Another crucial element in the durability of the plaque is the fibrous cap. Vulnerable plaques which have a higher likelihood of rupturing and causing acute coronary syndrome are covered with thin fibrous caps. Komukai et al. Conducted the EASY-FIT (Effect Of Atorvastatin Therapy On Fibrous Cap Thickness In Coronary Atherosclerotic Plaque As Assessed By Optical Coherence Tomography) trial compared the width of fibrous cap in 70 patients suffering from unstable angina who were given atorvastatin 5 mg per day versus the individuals prescribed 20 mg per day [24]. Fibrous cap thickness declined more in the higher statin dose group, which was linked to lower serum LDL and high sensitivity CRP levels.

### Reduction in inflammation

The manifestation of inflammation within the arterial plaque is well documented in people with well-established atherosclerosis or risk factors for atherosclerosis using FDG PET imaging. FDG uptake was reduced in a dose–response manner following treatment with high dose of atorvastatin, a shift apparent as quickly as four weeks post treatment commencement. The processes behind statin's anti-inflammatory response are assumed to be associated to their pleiotropic properties. Statins suppress the formation of reactive oxygen species (ROS) and the emission of pro-inflammatory cytokines such as TNF-alpha, interleukins [25]. As a result, this leads to reduction in monocyte recruitment, and therefore the plaque progression is halted. Also, there is shift towards the second phenotype of macrophage which further results in plaque stability.

## Impact of statins on different body organs

### Liver

The favorable effects of statins on the liver have been proven in numerous research. They appear to minimize the chance of developing progressive fibrosis and decompensating cirrhosis [26, 27]. Statins anti-carcinogenic characteristics, such as suppression of angiogenesis and activation of apoptosis, have been found to be beneficial in the treatment of hepatocellular carcinoma. Also, they are efficient in inhibiting the multiplication of hepatitis C virus and thus, preventing the infection from spreading. Furthermore, if the histology was utilized as an outcome metric, they supported the liver to perform its function in individuals suffering from non-alcoholic steatohepatitis(NASH) or non-alcoholic fatty liver disease (NAFLD).

In a randomized trial by T Foster and colleagues, it was observed that allocating patients to a combination regimen of vitamin E 1000 IU, vitamin C 1 g and atorvastatin 20 mg reduced their relative risk of developing hepatic steatosis by 71% with a baseline NAFLD as compared to placebo [28]. Additionally, statin treatment was discovered to reduce the incidence of cardiovascular events in people with elevated liver enzyme (AST, ALT) markers.

Statins have an impact on other markers and cardiovascular risk factors, like arterial hardness, albeit additional research is required to put these results into reality [29, 30]. Statins implicate all these hepato protective actions via multiple mechanisms which include reducing the abundance of bile acids which are thought to have a role in pregnane X receptor activation as well as alfa and gamma peroxisome proliferator-activated receptor of monocytes and macrophages, blocking hepatocyte signaling of on hepatic satellite cells and cytokines production [31], inhibit the RhoA/Rho-kinase pathway in HSCs and maintain endothelial and Sinusoidal function by KLF2 induction, which ultimately inhibits the activity of satellite cells. In human investigations, statins were found to have favorable effects on steatosis, which is the precursor to inflammation and liver fibrosis [32]. According to Chomphupan, a low dose of Simvastatin (10 mg) is helpful in intercepting stroke in type-2 diabetes patients [33]. Additionally, through controlling vascular tone through pathways like RhoA/Rho-kinase and nitric oxide, statins also serve to lower portal pressure.

## Kidney

Except for rosuvastatin and pravastatin, the majority of statins are processed in the liver, maintaining the lipid levels in patients of chronic kidney disease (CKD) is strongly advised and thus lower the incidence of cardiovascular diseases in chronic kidney disease. This relationship is termed as cardio renal syndrome [34]. There are contradictory evidences about the safety and efficacy of statins in individuals with kidney disease, mulitple randomized trials and studies have depicted that use of statins is not linked to deterioration of renal function but may have a Reno protective effect [35]. Contrarily, the risk of CVD mortality in CKD patients is cut by 12% when Ezetimibe and Simvastatin are coupled [36]. Lipid independent anti-inflammatory characteristics of statins are well renowned, which help them protect against acute kidney injury after a CVD procedure by diminishing the circulatory levels of IL 1,6 and 8 and C reactive proteins whereas the levels of IL 10, anti-inflammatory mediator were elevated [37]. Apart from preventive and curative effects of statins, they also have prophylactic effects. Statin usage prior to surgery is anticipated to reduce the risk of renal insufficiency in individuals undergoing heart surgery [38, 39]. The anti-inflammatory effects or the reno-protective of statins is due to the over-expression of KLF4. Therefore, statin use offers protection against acute renal illness in a variety of circumstances (contrast medium induced acute kidney injury) [40–42].

Although studies showing opposite results are also present. Perioperative statin usage has been shown to have little to no influence on postoperative adverse renal outcomes [43]. Furthermore, treating patients with acute renal injury who had sepsis or an accompanying respiratory distress syndrome with Rosuvastatin did not result in a beneficial response and may have worsened the situation [44].

Taking statins while a patient is receiving critical care is also associated with a general decline in the need for renal replacement treatments and mortality [45]. In a recent in-depth investigation, Nayan pointed out that statin use among kidney cancer patients is associated with greater survival rates [46].

Summing up all the evidences, it is understood that statins are effective in preventing acute kidney injury when used preoperatively but had no protective effect when used post operatively.

## Nervous system

Statins are potentially suspected to have an impact on the neurological system. They have been demonstrated to suppress the brain's inflammatory response. Simvastatin (20 mg/kg) has been shown in animal tests to diminish depression-like behaviour by lowering hippocampal inflammatory cytokines such as TNFA, IL1B and IL6, hence alleviating lipopolysaccharide-mediated depression and inflammation induced stress [47, 48]. In a randomized trial, Salagre et al. demonstrated that the symptoms of depression can be greatly reduced when statins and anti-depressive therapy are used simultaneously [49].

Jenson et al. depicted in their study that patients with multiple sclerosis, which is a chronic inflammatory disorder, have benefitted from the use of statins. They also demonstrated through their analysis that statins impose beneficial effects in diseases like multiple sclerosis and optic neuritis but there use to treat the disease is not recommended [50].

A high dose of Simvastatin was shown to arrest the advancement of multiple sclerosis in the MS-STAT clinical trial. Furthermore, the deterioration in memory and frontal lobe function associated with multiple sclerosis has been observed to recover after therapy [51]. Statins neuroprotective properties have been well shown in both experimental as well as clinical investigations. Statins block dopamine depletion, prevent neuron degeneration, and improve locomotor performance, demonstrating their ability to pass the blood–brain barrier [52].

Also, statins were seen to diminish the advancement of Parkinson's disease, Alzheimer's disease and loss of cognitive function [53–56].

### Bone

The first report of a putative link between the skeletal system and statins was published in the year 1999. Statins can significantly increase bone development by promoting the formation of bone morphogenic protein-2 (BMP2), according to animal research [57].

In a cohort study, statin therapy was revealed to lower the probability of osteoporosis among both men and women. The statin's osteoprotective effect may be linked to the total dose and statin efficiency [58, 59]. A study demonstrates the dual actions performed by atorvastatin which includes the increase in bone formation, elevation of biomarkers of bone formation was seen in the serum. Apart from this, the bone reabsorption is also diminished [60]. Statins' osteoprotective effects are driven by a variety of mechanisms, including the stimulation of vascular endothelial growth factor and the suppression of osteoclast production [61].

Statins appear to play a pivotal function in suppressing apoptosis, increasing the lifespan of progenitor cells which in turn enhances their ability to repair organ activity [62]. Simvastatin has the ability to reduce periodontal infection, diminish inflammation in the temporomandibular joint, improvise alveolar and periodontal bone regeneration, cartilage repair and soft tissue grafting [63]. In the mesenchymal stem cell line D1 [64, 65], it also increases the mRNA expression of the osteogenic markers integrin ITGA5, ALP, Runx2, BMP2 and OC. Also, Statins can control periodontal disease by reducing the expression of matrix metalloproteinases and pro-inflammatory cytokines, according to a subsequent in vitro and clinical study [66].

Simvastatin or Atorvastatin treatment, however, promoted bone mineral density [67] but had no impact on 25 hydroxy vitamin D levels at various dosages over the course of a year. In conclusion, cohort studies revealed that, in a dose-dependent manner [68], statin use was associated with a decreased incidence of hip fracture, vertebral fracture in stroke patients, and osteoporosis [68].

### Lung

Chronic obstructive pulmonary disease, abbreviated as COPD threatens 380 million individuals globally, which make up 12% of adults over the age of 30. This condition is becoming more widespread, and by the year 2020 it will be the third greatest cause of death globally. Chronic inflammation and tissue remodelling in response to potentially dangerous particles or gases are two frequent mechanisms underpinning the onset and progression of COPD [69].

According to recent studies, lung remodelling and healing might result from prolonged and/or severe inflammation in the lungs, which is triggered by smoking and worsened by genetic predisposition. COPD still lacks efficient medications that alter disease progression and improve survival due to the complexity of communication networks that perpetuate chronic inflammation and tissue death [70, 71]. Based on numerous investigations, individuals with COPD who utilize statins—especially Fluvastatin and Atorvastatin—have lower levels of c-reactive protein, pulmonary hypertension, and mortality risks [72]. Others have examined the impact of low, moderate, and high statin adherence in older individuals with concurrent COPD and CVDs, demonstrating that the high statin adherence significantly lowers the risk of chronic obstructive pulmonary disease exacerbations.

The evidence supplied was insufficient to determine the association between statins and COPD development without taking CVD into account, despite the fact that CVD is regarded as a common concomitant illness in COPD patients. Furthermore, a new study shows that respiratory virus infections are a significant factor in the development of COPD. This research also lends weight to other studies' findings that statins may have antiviral and anti-inflammatory properties (down-regulation of cytokines) [73]. Studies have also been done to better understand the underlying molecular theories of COPD. These results suggest that the protein osteopontin (OPN) and the enzyme adenosine deaminase (ADA) may have opposing roles in the onset of COPD. OPN expression is elevated by enhanced adenosine signalling, which is brought on by ADA deficiency [74].

Statins have a suppressive effect on OPN in cancer cells, but very little is known about how they affect COPD patients. According to the study's findings, Simvastatin restored IL13-suppressed ADA activity, which led to the down-regulation of adenosine signalling and prevented the synthesis of OPN via the STAT6 pathway that is directly inhibited by IL13.

The study also came to the conclusion that IL13 inhibition may slow the progression of COPD by rectifying the imbalance between OPN and ADA. Additionally, there is some evidence to support the idea that inhibiting HMGCoA reductase and the mevalonate pathway may have a preventative effect on the development of lung cancer, particularly in COPD patients who have heightened pulmonary inflammation and a higher risk of developing lung cancer.

## Role of statins in other medical conditions

### Cancer

Statins are well known to block the HMG co A reductase, the key enzyme in the mevalonate pathway. Although, inhibition of this pathway leads to lowering of circulating cholesterol levels, there are also other aspects related to this. While this lowers blood cholesterol levels, the mechanistic components also influence cell signaling, which could have a significant effect on cell proliferation and, thus, cancer cells.

Ceasing the mevalonate pathway, leads to decrease in the abundances of isoprenyl groups, geranylgeranyl pyrophosphate (GGPP) and farnesyl pyrophosphate (FPP), that dramatically modulate the small signalling G proteins which have a role in cell migration, proliferation and survival pathways, and the effect of this mechanism is not just limited to cardiovascular ailments but also expands to other serious illnesses, especially cancer, where progression is dependent on increased migration, survival, and eventually proliferation. Reduced abundance of GGPP and FPP reduces cellular signalling from small G proteins, resulting in cell-mediated consequences.

Thus, after assessing all the major and pleotropic effects of statins, it could be inferred that tumor cells would be harmed if the statin could obtain access in the cells (as in lipophilic statins). It is well documented that cancer cells require more cholesterol and the components utilised in the production of cholesterol in order to sustain a high level of proliferation. Also, surprisingly it has been discovered that levels of circulating cholesterol were consistently lower in patients diagnosed with cancer, although elevated in the membrane of tumor demonstrating that the use of cholesterol by cancerous cells and tumors is a key aspect of carcinogenesis and maybe metastasis [75, 76]. As statins affect the initial stage of the mevalonate pathway, the potent clinical effects of statin medication may hamper cancer growth by limiting the synthesis of cholesterol or inhibiting the generation of isoprenoids. In addition [77], Statins also possess anti-inflammatory properties via suppression of pro-inflammatory cytokines production, which may inhibit carcinogenesis and metastasis [78, 79]. Furthermore, statins' ability to diminish prenylation may suppress signaling through carcinogenesis and cancer progression pathways [80].

## Inflammtory bowel disease

Statins may have more complex activities than only reducing cholesterol because of their immunomodulatory features [81] which include leukocyte infiltration of target organs, antigen-presenting function, inhibition of T-cell activation and gives a hope that these agents could be used for treating immunological disorders [82, 83]. Evidences support that statin have beneficial effects in inflammatory bowel disease, an immune mediated disorder, which encompasses the two most common types of this disease: ulcerative colitis (UC) and Crohn's disease (CD) [84–86]. In IBD patients, statins have been linked to a lower utilization oral steroid during the acute phase, as well as a lower disease activity index and inflammatory markers [87, 88]. Another study found a link between statins and a lower risk of new onset IBD [89], increasing the likelihood of use of statins as a preventive measure in high-risk people in the near future.

Till date only 2 studies have been conducted to identify the relation between use of statins and as a consequence, development of new onset IBD [90, 91]. The first retrospective case control study was conducted by Ungaro et al. With 46,665 controls and 9617 controls. The follow-up was done from January 2008 to december 2012.The study depicted that in both UC and CD, statin use was found to be protective against new-onset IBD. With the exception of lovastatin and rosuvastatin in UC, statin usage was correlated to a lower threat of new-onset IBD. There was a safeguarding effect which was consistent amongst different intensities of statin, and this effect was more strongly observed in individuals aged 60 or older at the time of IBD diagnosis. After controlling for a number of confounders (such as antibiotics, hormone replacement therapy, non-statin lipid medications, CV disease, lipid disorders, diabetes medications and anti-hypertensive), statin use was found to have a similar protective effect across the entire cohort.

Khalil and colleagues conducted the second study, which was a retrospective cohort analysis with 43,438 individuals and a follow-up period from October 2005 to March 2015. Statin use was not linked to an increased risk of developing IBD in this study. In this study, 13,626 statin users and 29,811 non-users were included, and 224 and 370 people developed IBD, respectively. In the propensity score-matched sample of 12,684 participants, 93 and 92 people with IBD were diagnosed, respectively (6342 statin users and 6342 non-users). In the no-Charlson comorbidity cohort, there were 73 and 266 people with IBD, respectively (5761 statin users and 24,366 non-users).

### Statins as a putative IBD therapy

Apart from prevention, some studies also explain the use of statin for treatment of IBD. Treatment with statin was linked to an 18% reduction in the rate of steroid initiation in a retrospective cohort analysis of 11,857 IBD patients although these results were obtained only with atorvastatin in patients with UC and not in CD.

Also, a randomized control trial supported the results of these studies and has shown promising results. In this trial, 36 patients were subjected to 40 mg atorvastatin for a period of 24 weeks. After following for this time period, these patients underwent Seo assessment and the findings suggested that there had been a significant decrement in the Seo index in individuals on atorvastatin when compared to placebo. Also, there are other studies which support this finding [92, 93].

## Dementia

Previously conducted studies stipulated that statin might elevate the risk of dementia but present studies do not agree with it. Nowadays, multiple studies have been conducted to see the correlation between them most of them conclude that the statins lead to abatement of risk of dementia, Alzheimer's disease and non-Alzheimer dementia similarly in both the genders men as well as women.

Dementia, rather than being an individual disease is a clinical syndrome, in which there is a progressive retardation of a person’s mental ability. The main clinical sign of dementia is a progressive reduction in cognition. Majorly, dementia is of two types degenerative dementia and nondegenerative dementia. The first type, i.e., Degenerative dementia consists of Parkinson’s disease, Alzheimer's disease, Louis’s dementia, Huntington’s disease and frontal–temporal dementia, whereas nondegenerative dementia majorly consists of space occupying lesions, traumatic brain dementia and vascular dementia [94]. To improve cognitive effects and symptoms, there is no particular curative therapy for degenerative dementia [95].

## Conclusions

Statins have been always known and used for the treatment of elevated cholesterol level in the cirulation, but surprisingly there is a wider aspect to this. Apart from lowering the LDL-C in circulation, statins have shown to have cardiovascular and extracardiovascular benefits like anti-inflammatory effects, plaque stabilization, reducing platelet aggregation and adhesion, diminishing oxidative stress and improved vascular tone. Also, it showed positive effects in treatment of life threatening diseases like dementia, cancer, chronic inflammatory diseases like multiple sclerosis and optic neuritis, inflammatory bowel disease, COPD, non-alcoholic fatty liver disease and non-alcoholic steatohepatitis. This is a sign to explore the statins not only for prevention and treatment of cardiovascular diseases but also for other life threatning diseases and statins could prove to be a boon in the treatment of such diseases. Hence, more trials should be conducted to have a better picture so that statins could be added in standard treatment guidelines for the above mentioned diseases.

## Data Availability

Not applicable.

## Abbreviations

ADA: Adenosine deaminase
BMP 2: Bone morphogenic protein-2
CVD: Cardiovascular disease
CKD: Chronic kidney disease
COPD: Chronic obstructive pulmonary disease
CAD: Coronary artery disease
CD: Crohn's disease
FPP: Farnesyl pyrophosphate
GGPP: Geranylgeranyl pyrophosphate
IBD: Inflammatory bowel disease
IL: Interleukin
LDL-c: Low density lipoprotein cholesterol
NO: Nitric oxide
NCDs: Non communicable diseases
NAFLD: Non-alcoholic fatty liver disease
NASH: Non-alcoholic steatohepatitis
OPN: Osteopontin
PSA: Prostate-specific antigen
ROS: Reactive oxygen species
SMC: Smooth muscle cell
TNF: Tumor necrosis factor
UC: Ulcerative colitis
WHO: World Health Organization
